# The research relationship: participant perspectives on consent in biobanking

**DOI:** 10.1186/s12910-025-01199-0

**Published:** 2025-04-12

**Authors:** Rachel Thompson, Kate Lyle, Gabrielle Samuel, Jo Holliday, Fenella Starkey, Susan Wallace, Anneke Lucassen

**Affiliations:** 1 University of Oxford, Oxford, UK; 2 King’s College London, London, UK; 3 UK Biobank, Stockport, UK; 4 University of Leicester, Leicester, UK

**Keywords:** Consent, Biobanking, Ethics, Research-relationship, Research-relationship, Governance, Tissue, Data

## Abstract

**Background:**

This paper examines challenges associated with the governance of large-scale biobanks. As the collection and interrogation of population-scale data is increasingly positioned as a route to new understandings of health and disease, large-scale biobanks are becoming essential elements of research infrastructure. However, their longitudinal nature presents challenges for governance, particularly in relation to consent. Typically, participants agree to specific activities at recruitment, but evolving technologies make it difficult to anticipate future research applications at this time. Using a case study from UK Biobank, we demonstrate how trying to reconcile new research activities with old consent risks overlooking critical ethical issues —particularly how the proposed activity aligns with participants’ understanding and expectation of biobank research.

**Methods:**

We conducted focus groups with UK Biobank participants using individual and group exercises to explore their views on consent and different types of research on their samples and data.

**Results:**

Our findings show that participants locate responsibility for research decisions with the biobank, rather than seeking control through their consent. They perceive their consent not as a one-off agreement but as the `opening act' for a research relationship with the biobank that can be continued through communication.

**Conclusions:**

Focussing on the ongoing research relationship -and the practices that sustain it- is more important than the specific wording on consent forms signed at recruitment. We argue this will be more effective in meeting participant expectation as well as supporting ethical research.

**Supplementary Information:**

The online version contains supplementary material available at 10.1186/s12910-025-01199-0.

## Introduction

As the collection and interrogation of population-scale data is increasingly positioned as the route to new understandings of health and disease, large-scale biobanks have emerged as essential elements of research infrastructure. While these repositories are designed to collect, store, and manage bio-samples and data to facilitate a broad range of research applications over time, their longitudinal nature (often extending over decades) presents challenges for governance. Health research governance typically relies on a one-off consent model, where participants agree to specific research activities at the outset. Yet, as technological advancements continue to enable new kinds of data collection and analyses, it may not be possible to foresee all future research applications at the time participants join a biobank. This tension has prompted questions about the validity of participant consent for these future uses [1, 2]. The inadequacy of the one-off consent model in comprehensively addressing ethical concerns in research is reflected in the use of qualified consent approaches such as ‘deferred’, ‘broad’ or ‘dynamic’ consent [3]. Although these approaches have been proposed as solutions which allow participants to update their preferences and decisions about research activities over time, determining if and when additional consent is necessary can remain ambiguous [4, 5, 6].

This paper argues that rather than exploring how governance requirements might be satisfied through different qualifiers for consent, the more pertinent issue is to understand what consent means to participants. This became evident recently in our capacity as researchers for the UK Biobank Ethics Advisory Committee (EAC) when UK Biobank proposed to access tissues held in participant medical records. As we describe in the case study below, discussions between UK Biobank and the Human Tissue Authority (HTA) about whether access to stored tissue was covered by existing consent focused on reconciling the proposed activity within the specific wording of consent agreed to by participants some 15 years earlier. Concerned that this approach fails to adequately address the ethical issues at stake —namely how the proposed activity aligns with participants’ understanding and expectation of biobank research– we conducted qualitative research with biobank participants to explore their perceptions of consent. Our findings reveal that participants view their engagement with UK Biobank not as consenting to specific activities, but as entering into a relationship where the organisation will manage research ethically on their behalf. In this way, we argue that focusing on the wording of consent may jeopardise important research with longitudinal cohorts that could align with participants’ expectations and aspirations for, but not with the specific language used in consent forms. Instead, we suggest biobanks should focus on building a maintaining a research relationship with participants in which consent is the opening act for ongoing relations.

## Case study: UK biobank

UK Biobank is a long-term prospective health research study set up in 2006 to provide a repository of samples and data for use in research exploring the prevention, diagnosis and treatment of serious illnesses. Between 2006 and 2010 half a million people aged 40–69 years were recruited across Scotland, Wales and England. They provided biological samples and detailed biographical and lifestyle information during recruitment and agreed to allow ongoing access to their medical records. Additional data were collected in subsequent initiatives, for example multimodal imaging, health questionnaires, whole genome sequencing and biochemistry markers [7]. All UK Biobank data are de-identified and made accessible to accredited researchers globally, following a research access approvals process.

During recruitment UK Biobank participants gave consent for their medical records to be accessed over time so that their ongoing health and disease status could be linked with information they had provided to UK Biobank. The contents of medical records vary considerably depending on participant (ill) health trajectories; for some participants samples of bodily tissue from clinical investigations (such as a biopsy) will have been retained as part of their records.

Since recruitment of the cohort, UK Biobank has accessed some data from participant medical records and linked these to the analyses of samples and data collected from participants at recruitment. Other data have been more difficult to access —usually because samples retained as part of medical records are still stored in physical form in NHS pathology laboratories and are not in an easily accessible digital format.

UK Biobank sought clarity as to whether participant consent allowed access to tissue samples stored as part of NHS pathology records for health-related research. The Human Tissue Act [8] governs research access to bodily tissues in England and Wales and so the UK Biobank Ethics and Governance Council^1^ contacted the Human Tissue Authority (HTA) for advice on how such samples might be accessed as part of UK Biobank data linkage to medical records. In support of this submission UK Biobank shared results from an internal survey they had conducted with their participants about understandings of various aspects of UK Biobank activity. Survey responses showed that some participants had not recalled their consent included access to medical records and that approximately half of respondents did not realise that their medical records might include tissue samples. However, the survey did not go on to seek participant opinions about these issues.

The HTA emphasised that the Act allows for exemptions to consent but explained that consent should be a positive act that ensures participants are not surprised by the use of their contributions. They drew on the internal survey responses and responded that participants might indeed be surprised by such use given that only half of the survey cohort thought that their medical record might contain stored tissues. The HTA also scrutinised the wording of consent at recruitment and considered that because there was no explicit mention of tissue samples further consent might be necessary.

UK Biobank’s EAC recognized these discussions as an opportunity to explore participant views about their consent and research access to samples in their medical records. This was in part because seeking further consent from half a million people would mean diverting considerable resources to the endeavor, when consent had already been provided to ongoing access to medical records.

## Methods

Our aim was to explore UK Biobank participant perspectives on consent and its role in the ethical conduct of biobank research with a particular focus on access to tissue samples within medical records. We conducted 12 focus groups with 94 UK participants which ran for approximately 90 min each and asked participants to engage in activities that explored the themes of collecting, storing and research use of samples and data from participant medical records^2^. For example, we used a vignette about a skin biopsy^3^ to discuss cellular tissue that might be stored following a procedure and then discussed in general terms the acceptability of UK Biobank access to health data and samples and the differences between them (a full schedule of activities can be found in appendix 1). Participants were brought together to discuss their responses. insert highlighted para here

We chose to run a series of focus groups, facilitated by one or more researchers, to enable interactive discussions that would allow for a nuanced and deeper exploration of the issues than a survey could. We incorporated facilitated activities to reduce the likelihood of those discussions being dominated by polarised opinions (9; 10). These activities were designed to explore both individual participant views and group interactions.

We conducted ten focus groups in person and two focus groups online (Zoom) from November 2022 to August 2023. Locations were chosen to achieve a geographic spread across the three nations represented in UK Biobank^4^. Venues were chosen to be near public transport and accessible. There were two locations each in Scotland and Wales, and six across England, representing the relative proportions of those populations in UK Biobank and ensuring a mix of location types as well as North and South per nation^5^. Online focus groups were added to enable access for those unable to attend a local group.

Once locations had been provisionally chosen, participants were selected by postcode area, invited to express interest and provided with a participant information sheet and a consent form via UK Biobank’s participant website. UK Biobank provided EAC researchers with contact information for those who had expressed interest, then researchers sampled potential participants, aiming to maximise the diversity associated with their demographic criteria^6^ across the study. Where there was variation in demographic characteristics, we prioritised outliers e.g. higher deprivation scores, age and lowest educational level for invitation. However, despite these efforts, our final study sample demographics closely mirrored the overall UK Biobank population composition.

A member of UK Biobank staff was present at each focus group to facilitate an open question and answer session following the discussions. This was requested by UK Biobank in order to respond to any technical or procedural questions from participants, and as an opportunity to engage face-to-face following cessation of UK Biobank in-person events during COVID restrictions.

A total of 94 participants took part in focus groups —87 in-person and seven online —and their demographic information (categorised by UK Biobank and received by researchers during recruitment) is reported in Table 1. Table 2 shows the number of participants per focus group.

**Table 1:** Participant characteristics

Category	Sub-category	
**Age Band**	Under 70	70–79
Participants per sub-category	49	45
**Deprivation Index Score**	Low	Medium
	58	36
**Ethnicity**	White	Non-White
	90	4
**Highest Qualification**	Secondary school or Technical	College or University
	29	65
**Sex**	F	M
	46	48

**Table 2:** Participants per focus group

Location	Number of participants
Oxford	8
Inverness	9
Glasgow	8
Lancaster	6
Swansea	9 (+ 1*)
Plymouth	7
Bangor	11
Leeds	8
London	10
Norwich	11
Online 1	4
Online 2	3
**Total**	**94**

* One participant attended with a caregiver who supported them to interact with the group

Some limitations of the method include that focus groups are known to favour those who are more confident interacting in social situations and expressing themselves verbally [9]. It is also likely that participants were amongst the more engaged participants of the biobank cohort and therefore potentially more positive about their experiences with UK Biobank [10]. Travel expenses were refunded rather than pre-paid and we did not remunerate participants for their time (in line with UK Biobank policy). Online groups had a lower take-up from participants than in-person groups and higher attrition. To help navigate some of the known challenges of online groups, such as information processing and group dynamics [11], we offered pre-session technical and access support and sent materials to participants ahead of time.

Each focus group was audio recorded and transcribed for analysis, with identifiers removed at transcription stage and then de-identification manually assured by a researcher. We conducted thematic analysis of focus group transcripts applying both an inductive and deductive approach to coding. Our analysis was informed by Braun & Clarke [12] and we used NVIVO qualitative data analysis software. Two researchers independently read the first three transcripts then collaboratively assigned high level codes or themes. Following this, one researcher coded all focus group transcripts using a combination of those themes (deductive) and generating new ones (inductive) as they engaged with the data in detail and shared their coding with a second researcher for assurance. The results of the analysis are reported in the findings section below.

## Findings

In discussing their involvement in UK Biobank, focus group participants made little distinction between data and samples held as part of their health record, believing that they had given permission for access to both during the recruitment consent process. Indeed, as we describe below, the details specified in their original consent were not significant to them, and they wanted UK Biobank to have access to record contents without needing to provide additional consent. As we go on to show, they considered that requests for further consent would contradict their altruistic reasons for participation and explicitly located responsibility for ongoing ethical work with the biobank.

### Data and samples in medical records

While nearly all focus group participants understood that data about them was included in their medical record, not all knew that tissue might be retained as part of that record. Some participants, particularly those with experience in healthcare or research, clearly understood that their tissue samples were included in records, while others were unsure or had never considered it:

…I’m unsure as to what actually found its way onto…, the NHS record.….

*Never really addressed my mind to the subject…*.

*‘Yeah*,* can I just echo that*,* I was exactly the same… never thought about it until you asked…* ‘.

Irrespective of their awareness, participants supported UK Biobank accessing such samples, where they existed. They did not distinguish between samples and data in their medical records and, importantly, did not consider they required separate consent:

‘…*as far as I’m concerned*,* whatever has been kept and recorded*,* whether it’s information*,* pictures*,* or that little bit of bone marrow*,* it’s all stuff that’s on my medical record and I’m happy for Biobank to access it if it’s going to be useful*.‘

Interestingly, not all participants remembered the details of what they had given consent for in terms of access to their health record yet, despite this, they did not wish to be asked for further consent for samples:

Participant A: *I knew that all these things you list form part of my medical record. I must admit I don’t recall giving Biobank permission to access it.*

*Participant B: I think it was asked in the original onboarding.*

*Participant A: I am certainly not saying that they didn’t ask*,* I just don’t remember.*

*Participant C: I can remember being asked.*

*Participant D: Like you*,* I’d forgotten that they asked but it doesn’t bother me*.’

Requesting further consent to access stored tissue was regarded as unnecessary because participants felt they had already granted access to the full medical record.

*To be quite honest*,* I find this* [being asked about consent] *rather irrelevant. I signed up to give all the information they want and I don’t care. I don’t want to have to think about it or be asked…*.

Moreover, they saw drawbacks to seeking further consent, including the extra resources and effort that would be required from UK Biobank:

‘… *and just to add that efficiency requires this*,* otherwise if you were giving permission for this and not for that*,* the whole thing would cost ten times as much.* ‘.

There were concerns that repeatedly asking participants for consent to use samples and data could potentially diminish the value of the consent process, and turn it into a tick box exercise.

*‘…but if you ask every time*,* people will get bored and just go yeah it’s OK’*.

Participants also discussed the potential for attrition from the UK Biobank cohort if there were repeated requests for consent. Their concern was that participants might withdraw due to inconvenience, rather than because of objections to potential uses of samples and/or data. Participants viewed UK Biobank as capable and better placed to determine ethically permissible uses of their data and samples than they would be as individuals. Essentially, they saw their role as enabling research and UK Biobank’s as managing research decisions on their behalf. They attributed this delineation to their lack of detailed knowledge about research approaches and ethical issues, as well as their confidence in UK Biobank’s expertise and systems:

‘*Yeah*,* choose your researchers carefully and let them get on with it. Obviously*,* it’s their [UK Biobank] decision whether they authorise research into* [a controversial issue] *… and there will be considerable protocols in place to do that. But again*,* leave it up to them*,* it’s their job.‘*

Participants expressed concern that seeking further consent would impede UK Biobank’s ability to facilitate research in a timely manner:

‘Don’t tie their hands with forcing them to have to come back to you to get your permission for use of your information… That does tie people’s hands quite considerably, I think, and devalues the purpose for which something like Biobank is set up in the first place.‘

Participants described their eagerness to contribute towards cumulative and widespread (research) benefits for all:

‘*For me it’s giving freely and also the scale… So*,* it’s recognising that*,* it’s given freely*,* but ultimately*,* it’s got a greater good*,* you know*.‘

‘*Generally*,* the pace of progress with research has just seemed to get faster and faster…And so that actually was part of the motivation that we talked about*,* our contribution is actually*,* you know*,* it kind of adds more and more weight over time as you can see it being used more and more*,* and more widely around the world as well.‘*

As we have shown, participants did not make distinctions between data and samples with respect to needing further consent, nor did they place significant value on the specifics of the consent process. Instead, participants were interested in how biobank practices and procedures operate to protect their interests without wishing to control access or determine uses themselves.

### Research access to medical records

Participants discussed the nature and risk of research activities including considerations around privacy, and identifying the boundaries of permissible access to their data and samples. Interestingly, discussions of risk were mostly centred not on themselves, but on hypothetical ‘other’ participants.

Participants’ discussions about access to health data and samples for research purposes largely centred on the potential for stigma and distress. Historical scandals around storage of tissues, such as Alder Hey [13] were used as illustrative examples. They drew distinctions between kinds of information, noting that some could be highly sensitive for certain individuals. Data or information related to pregnancies was raised as an example:

‘*I think there may be things*,* say you’d had a miscarriage or something like that*,* I know there has been stuff about babies*,* parts of unborn children*,* things like that*,* that can upset people*.’ [refers to both stored tissue and data about such events that would be held in the health record].‘

They also discussed the potential for individuals to be identified from the data, even when direct identifiers (e.g. name, date of birth, specific locations) had been removed. They emphasised the importance of safeguarding individuals who may be particularly vulnerable to exploitation of their information, again focusing on concern for others:

…*I think of this kind of sensitive stuff* [record contents] *maybe… being used against in some way…*.

Participants discussed how historical means of recording health related events might be problematic given that medical records may contain information spanning a person’s entire life. They considered older information relating to sensitive diagnoses, and reflected on changes in how information is recorded over time. For example, these participants discussed the possibility of subjective opinions from healthcare professionals being part of their record:

*It could be*,* I don’t know*,* a sample taken*,* a cervical smear taken of a young person*,* and in this written report it says shows promiscuity or something like that… Hope it wouldn’t happen now*,* but 20 odd years ago…*.

*‘If… I’d had a psychotic episode*,* [notes]…I would be less comfortable with it staying on my record for 30 years and* [imagine that] *I’ve never had another one but suddenly a doctor or researcher looks back and says… Do you see what I mean?’*

Throughout their discussions about risk and permissible research access to medical records, participants considered the potential research value for current and future generations to be significant:

‘*I do think it’s really*,* really precious to keep this kind of [healthcare] information*,* you can see the layering of it*,* and how much information you can give about you*,* about my son*,* about my son’s children*.‘

Participants discussed the factors that enable confidence in UK Biobank to manage access and associated risks, and below we consider their collective role in establishing and sustaining the participant-biobank research relationship.

### Building a sustainable relationship

Participants’ accepted risks associated with participation because they had a shared understanding that responsibility for the control of data and samples resides with UK Biobank rather than being governed via participant consent:

‘*Well*,* it’s logical*,* I mean*,* if you sign up for a project like Biobank in the first place*,* it would*,* in my opinion*,* be somewhat illogical to expect to be contacted at every verse end for consent for this*,* consent for that*,* consent for the other. Because you as an individual*,* do not know anything about the conduct of research projects*,* the moral and ethical constraints which are on them anyway. So… it would just be totally impractical.‘*

They described a series of factors that have fostered their confidence in UK Biobank’s ability to act ethically in respect of research access to participant data and samples. First, a sense of satisfaction at being able to help with research had been reinforced through interactions with and knowledge about UK Biobank and the research it facilitates. Participants described how their confidence in the biobank’s ways of working has developed through experiences including the recruitment process, research communications and reporting about UK Biobank in the global media. Participants also described the absence of significant criticism of UK Biobank in the wider media since they agreed to participate. They noted reporting of research findings supported by UK Biobank data, which emphasised the positive impacts of research utilising their samples and data:

‘*I think it’s also significant… that there hasn’t been any scandal about Biobank that I’ve noticed since it’s begun*,* which suggests that these protocols are being properly implemented.*‘

They discussed informational materials from, and their desire for ongoing communication with, UK Biobank. In particular, they mentioned the repeated messaging they received from UK Biobank regarding ethical processes. As participants explained:

… *we’ve been reminded of the ethical setup almost every time*,* if not every time*,* that we’ve had any contact with Biobank*,* and it gets drummed into us.*

‘*…It was 10–15 min of it at the start here and all of the bumph that we got through email*,* half of that was about that [ethics] sort of thing. So*,* we trust it… if there were breaches in that*,* then there would be a problem*.‘

Many participants described the messaging around ethical conduct as fostering their trust in the brand of UK Biobank as well as the individuals with whom they interacted. They felt able to rely on organisational processes to ensure the proper use of data and samples, and viewed these processes as demonstrations of trustworthiness; ethical and integral to their confidence in the research process:

‘*Again*,* it’s all that…confidence in the ethical base of what’s happening.*‘

Participants mentioned ethics committee oversight as an example of such a process:

‘*I’m happy for them to access it [*the data/samples*]*,* provided that there is a review of the purpose*,* the outcomes*,* because things change… So*,* if there’s Ethics Committee review… which I know they typically do*,* then I don’t have an issue*. *But it’s about processes being in place*,* rather than just blind trust because things evolve*‘

They also highlighted reminders about the option to withdraw from research, emphasising the importance of ethics-related communications in feeling confident about their choice to remain involved:

‘*I think every communication tells us we don’t have to do this* [take part]. *And including today…. So yes. I mean I haven’t thought about it since I signed up but*,* of course*,* I’m sure we signed up to give everything*,* unless you’re going to do a reveal at the end*.‘

‘*…so*,* when originally each person signed up to all of that and you gave your permission…. So*,* that people were clear as to what they were signing and giving their permission for. And then if*,* however down the line*,* you could withdraw or opt out*,* maybe.*‘

While views on frequency, type and methods of communication varied, there was a broad agreement that communications were an important relationship-supporting factor. Participants described the significance of communications including external media reporting, research-related interactions, and governance processes in fostering the relationship over time.

## Discussion

This research was initially prompted by UK Biobank’s inquiry into accessing tissue samples stored in medical records. Discussions between UK Biobank and the HTA were framed by regulatory requirements, and the HTA’s advice that new consent would be required was based on important distinctions between data and samples. This approach does not align with the intent of biobank participants’ consent, as described through our research. Even when participants were unaware that their stored tissue might be part of their medical record, they did not express a desire for further consent to access. Instead, they viewed their consent as part of a broader relationship with the biobank, wherein they expected UK Biobank to determine the appropriateness of research involving their medical records (samples and data).

The concept of a research relationship has been explored elsewhere, with Kraft et al. noting that *‘research relationships depend critically upon the willingness of participants to entrust some aspects of their well-being to researchers especially in the context of long-term or ongoing researcher–participant relationships.’* [14]. We extend this work to view consent as the initiator, or ‘opening-act’, for an ongoing relationship between participants and the research institution, rather than a singular activity through which all research uses will be defined. This research relationship is built and strengthened through ongoing demonstration of the biobank’s values in action, reinforcing participant perception of research as a collaborative project, underpinned by shared goals and values, and enabled through the research relationship. Our research suggests that as the relationship develops, ethical considerations typically assigned to formal consent processes may be able to be addressed and managed through reflexive governance and communication without the need for further consent.

The limitations of relying on consent to manage all ethical considerations in research are well-recognised and have prompted the development of ‘qualified consent’ approaches, such as dynamic consent, that aim to offer participants more control over research activities [15, 16]. However, our research indicates that participants are not seeking greater control over research access through consent; instead they want the biobank to demonstrate trustworthiness, and be able to trust it to manage this responsibility on their behalf. As such, efforts to resolve ethical challenges that rely upon modifying or qualifying participant consent are neither effective nor appropriate for longitudinal questions of participant data/sample use. Ethical matters may be more effectively taken care of through well-supported research-relationships, and resources should be directed towards cultivating such relationships throughout the lifecycle of the biobank. Cultivating relationships includes practising effective communication, responsivity and transparency of ethics and governance procedures.

In terms of the former, effective communication must include *how* participants’ contributions are employed, by whom, and how these contributions are benefitting others: participants joined UK Biobank primarily for altruistic motives [17] and wanted to see evidence of the societal value of the biobank. In terms of the latter, ethics and governance procedures need to be accessible, offering avenues for critical engagement, and providing ongoing involvement opportunities with participants, all of which must be carefully considered, responding to context-specific needs [17].

By focusing on building relationships through investment in these practices, biobanks can develop a robust and flexible relationship with participants that is sustainable and adaptable to changes in research capability and/or needs. However, this is not to suggest that new consent has no role in longitudinal research; certain types of novel research activity involving new investigations, may necessitate new consent. To prevent issues of consent ambiguity in the future, it is important that biobanks work with participants to identify which activities may require new consent, then determine how they should be managed. When new consent is required, it should not merely involve providing information to be accepted or rejected, but rather be a series of iterative and interactive communications [4].

The characterisation of consent as one part of a multifaceted research relationship between participants and a research biobank aligns with participant understandings of consent, and their expectations of how biobanks should enact their ethical responsibilities. By moving away from a focus on reconciling choices with the wording of consent documentation towards enacting participant understandings of consent, this approach can better accommodate the complexities inherent in longitudinal biobank studies, which often challenge regulatory conceptions of consent [18]. Importantly, this also avoids placing undue emphasis on individuals’ control through consent or permission-giving [19, 20, 21]. Therefore, this broader perspective on research relationships is better suited for navigating ethical issues in biobank research than reliance solely upon the documentation of consent.

## Electronic supplementary material

Below is the link to the electronic supplementary material.

Supplementary Material 1

## Data Availability

The datasets generated and/or analysed during the current study are not publicly available due to the conditions under which ethical approval was granted. Please contact the corresponding author(s) with any queries about the data.

## Footnotes

1 The EGC was disbanded in 2017. A new Ethics Advisory Committee (EAC) internal to UK Biobank was established in 2018.

2 See Appendix 1 for Focus group activity schedule and discussion prompts.

3 Exercises were developed in collaboration with UK Biobank. Skin biopsy was used as an example of a clinical investigation that could result in the retention of tissue as well as data in the healthcare record.

4 s Scotland, Wales and England.

5 ical investigation that could result in the retention of tissue as well as data in the healthcare record. ^3^ See Appendix 1 fo.

6 r Focus group activity schedule and discussion prompts with researchers: sex at birth, age band, deprivation index score, highest educational qualification, self-reported ethnicity (categorised by UK Biobank).
