# Supplementary material for: The research relationship: participant perspectives on consent in biobanking
Source: BMC Med Ethics. 2025 Apr 12;26:47. doi: 10.1186/s12910-025-01199-0 (PMC11992699; doi:10.1186/s12910-025-01199-0)
Supplement: Supplementary file 1 — Supplementary Material 1 [file 12910_2025_1199_MOESM1_ESM.docx]

# Appendix 1

**FOCUS GROUP DISCUSSION SCHEDULE:**

**8-10 participants for 1 hour 30 mins to two hours**

1. **Participant introductions (5 minutes)**

*To help participants feel comfortable*

To get to know one another a little better, we’re going to go around the group introducing ourselves using our first name only, along with one fact about ourselves, e.g. something you like or dislike; a food or an animal for example.

1. **General impressions (10-15 minutes)**

*To ‘warm up’ participants and understand their views and experiences of UK Biobank*

To start off with, and to warm ourselves up, I’ve popped a series of questions on the screen. I’d like you to work through them individually for 5 minutes. We’re then going to discuss them as a group. During the group discussion, we’ll share and reflect on each other’s views and experiences. It’s likely that you each have different views, experiences and beliefs, and that’s totally fine – we want to hear about them.

- What does the word ‘biobank’ mean to you?
- What samples and data do you think UK Biobank stores?
- Why did you decide to take part in UK Biobank?
- Now that you have been a participant for many years, have your reasons for taking part changed at all, and if so, how?

Prompts for discussion:

- Why do you have particular perceptions/views?
- Was there something that particularly stood out for you that didn’t for others?

1. **Activities (45-60 mins)**

We’re now going to do the main activity of this session. I’ll give you a brief introduction and you can ask any clarifying questions.

When you have a blood test taken as part of an investigation in the NHS (at your GP or in a hospital), the leftover sample is usually disposed of after the test is complete, but other types of samples may not be. For example, if you have a biopsy taken - perhaps a mole removed - then the skin (tissue) sample will be examined under a microscope. The microscope slide will be stored, and the rest of the mole might also be stored so that it can be re-examined in the future. Information about the sample - a description of the findings - is also stored, as may be a digital image of the microscope findings. The stored sample, the digital photo and the interpretation are all stored as part of your medical record.

As a participant in UK Biobank, you gave your permission for UK Biobank to access your medical and other health-related records – including any samples stored - so that information from these can be linked to samples and data you provided at UK Biobank recruitment as well as through other activities – such as imaging scans, questionnaires, and antibody tests – that you may have helped with since then.

In this activity, we’re interested in exploring whether you knew that the samples and associated information stored by the NHS are considered part of your medical record, if you think you have given permission for UK Biobank to access such specimen samples and/or the information associated with the samples, and, importantly, your views on this.

Your answers may depend on a particular context, and you might have different views within the group. We’re interested in these different contexts and views and encourage everyone to share their thoughts.

We’re going to do an activity to help us talk about this. You may do this activity individually, or in pairs if you prefer, and then we will discuss it. The purpose of the activity is to prompt you to think about different issues, so as you complete the activity, please do make notes of anything you want to bring back to the group discussion, and please don’t be afraid to draw on anything that is relevant – something you read somewhere, or watched (a film perhaps, or in a newspaper) - or an experience you have had that has helped to shape your view.

The activity involves imagining that you have been to hospital to have a small skin bump removed (under local anaesthetic).

Think about three stages of this investigation: (Image 1) the sample is taken and stored in the pathology laboratory, (Image 2) a tiny slice from the sample is examined under the microscope, and a photo is taken of it, and (Image 3) a report is written about the findings.

We are interested in whether you were aware that the specimen sample, data and/or microscope picture were stored as part of your medical record.

We are also interested to know whether you consider that you have given permission for UK Biobank to access these samples and/or data, and your views on this.

Facilitator explains that the activity is a viewpoint scale, and ask participants to choose where their view lies between numbers 1-9 and jot these down as we will be coming back to them and discussing them in more detail. Remind them to think about the reasons for choosing the number they choose.

My doctor took a biopsy from a mole on my skin and this biopsy has been store

d in the laboratory and linked to my medical record. UK Biobank would like to access a very small amount of this specimen.

**Image 1: Tissue (specimen) sample**


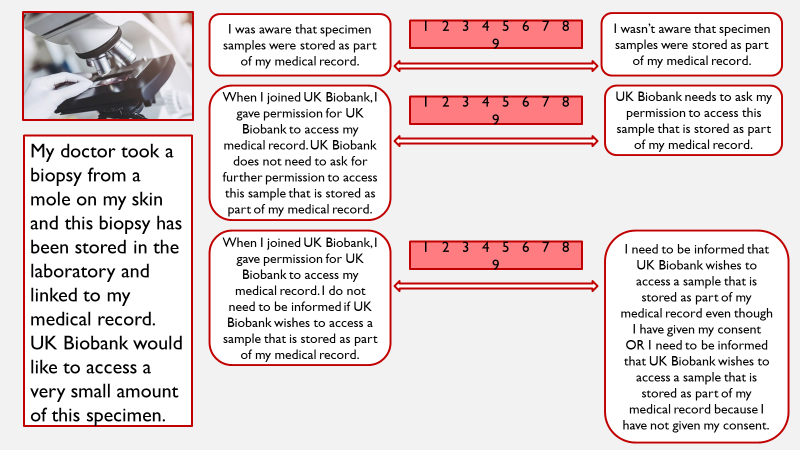


**Image 2: Pictures or images**


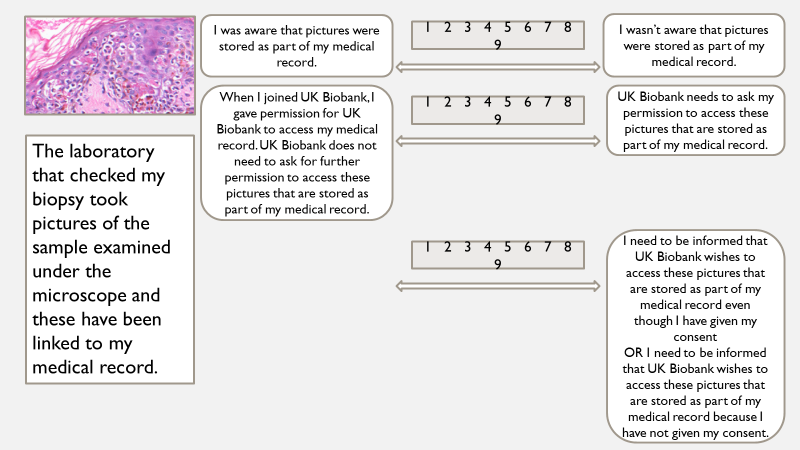


**Image 3: Report (findings)**


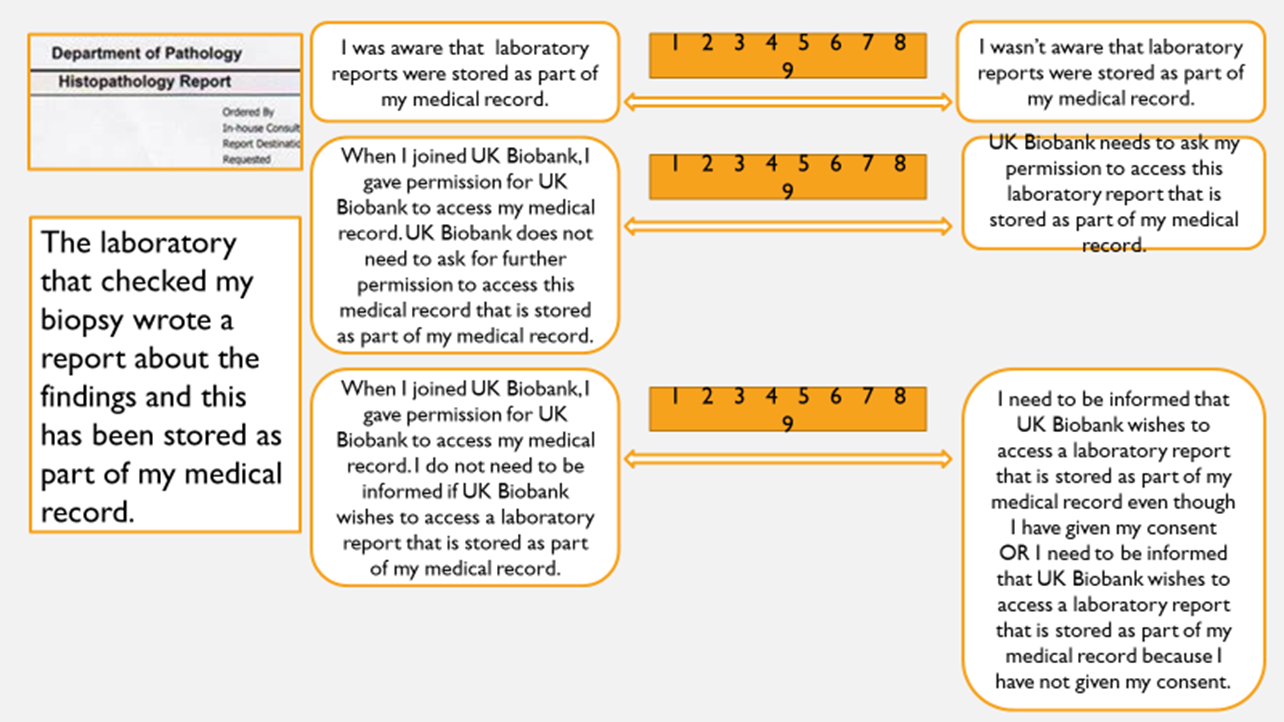


Facilitator asks people to feed back about how they scored the scenarios, and then redo the activity as a group during which the following prompts can be used as needed:

- How did you find this task? What was easy/hard about it?
- Why did you pick these scores - was it because of something you read or knew about, or something you experienced? How does that compare to others?
- What surprises you about where someone else has placed their score on the scale, and why?
- Are there any differences between the sample, report and pictures?
- Is the finite nature of a sample relevant to any differences? Does knowing only a tiny fraction is needed make a difference?

**Prompts if needed during the discussion:**

- If you were someone who wanted UK Biobank to ask for extra permissions, can you say something about how you think that should be done?
  - What risks or barriers might be associated with doing this?
- Do you want to be asked to sign a new consent form, for example?
  - Would you be happy to complete an online consent form?
    - What might be the advantages and disadvantages of such an approach?
- What does ‘being informed’ mean to you when you say you want to be informed?
- How much information would you want to receive, how would you like to receive it and when would you like to receive it?
  - What risks or barriers might be associated with doing this?
- Why is this important to you/not important to you?
- Does it matter that you might not be aware that specimens are being stored in your record or that UK Biobank is accessing them?
- Are there any ‘lines in the sand’ where you think it is NOT okay for UK Biobank to access information about participants’ medical care? Can you think of any specific conditions or contexts?
- Do your answers depend on *when* or *where* the sample/data/tissue was taken, or for which disease/condition the sample was taken? What about if participants have died? What about if UK Biobank has no means of contacting an individual?
- When the sample is stored, the hospital can access it for training or education without asking your permission.
  - Did you know this?
  - How do you feel about that?
  - What, if anything, do you think may be different between this kind of access (to samples) and the sort of access that UK Biobank has?

1. **Close (5 minutes)**

I want to thank you all for your participation and ask if anyone has any questions. Also, if you feel uncomfortable asking questions in front of the group, please feel free to email me over the next few days or stay in the room/on the call if you would like to discuss further.

We now have an open Q&A with a member of UK Biobank staff, which is your opportunity to ask anything you would like to about your participation, ongoing research or UK Biobank in general.
